# Efficacy of a Marine Bacterial Nuclease against Biofilm Forming Microorganisms Isolated from Chronic Rhinosinusitis

**DOI:** 10.1371/journal.pone.0055339

**Published:** 2013-02-18

**Authors:** Robert C. Shields, Norehan Mokhtar, Michael Ford, Michael J. Hall, J. Grant Burgess, Mohamed Reda ElBadawey, Nicholas S. Jakubovics

**Affiliations:** 1 School of Dental Sciences, Newcastle University, Newcastle upon Tyne, United Kingdom; 2 Advanced Medical and Dental Institute, Universiti Sains Malaysia, Penang, Malaysia; 3 Microbiology Department, Freeman Hospital, Newcastle upon Tyne, United Kingdom; 4 School of Chemistry, Newcastle University, Newcastle upon Tyne, United Kingdom; 5 School of Marine Science and Technology, Newcastle University, Newcastle upon Tyne, United Kingdom; 6 Department of Otolaryngology and Head & Neck Surgery, Freeman Hospital, Newcastle upon Tyne, United Kingdom; 7 Otolaryngology Department, Tanta University, Tanta, Egypt; University of Malaya, Malaysia

## Abstract

**Background:**

The persistent colonization of paranasal sinus mucosa by microbial biofilms is a major factor in the pathogenesis of chronic rhinosinusitis (CRS). Control of microorganisms within biofilms is hampered by the presence of viscous extracellular polymers of host or microbial origin, including nucleic acids. The aim of this study was to investigate the role of extracellular DNA in biofilm formation by bacteria associated with CRS.

**Methods/Principal Findings:**

Obstructive mucin was collected from patients during functional endoscopic sinus surgery. Examination of the mucous by transmission electron microscopy revealed an acellular matrix punctuated occasionally with host cells in varying states of degradation. Bacteria were observed in biofilms on mucosal biopsies, and between two and six different species were isolated from each of 20 different patient samples. In total, 16 different bacterial genera were isolated, of which the most commonly identified organisms were coagulase-negative staphylococci, *Staphylococcus aureus* and α-haemolytic streptococci. Twenty-four fresh clinical isolates were selected for investigation of biofilm formation *in vitro* using a microplate model system. Biofilms formed by 14 strains, including all 9 extracellular nuclease-producing bacteria, were significantly disrupted by treatment with a novel bacterial deoxyribonuclease, NucB, isolated from a marine strain of *Bacillus licheniformis*. Extracellular biofilm matrix was observed in untreated samples but not in those treated with NucB and extracellular DNA was purified from *in vitro* biofilms.

**Conclusion/Significance:**

Our data demonstrate that bacteria associated with CRS form robust biofilms which can be reduced by treatment with matrix-degrading enzymes such as NucB. The dispersal of bacterial biofilms with NucB may offer an additional therapeutic target for CRS sufferers.

## Introduction

Chronic rhinosinusitis (CRS) is one of the most common upper respiratory tract diseases, affecting approximately 10% of the adult European population [1]. Rhinosinusitis is an inflammation of the paranasal sinuses that is almost always accompanied by inflammation of the nasal airway and is classified as ‘chronic’ if it lasts at least 12 consecutive weeks [2]. The symptoms of CRS include blockage or congestion of the nasal passages, nasal discharge, facial pain or pressure and/or a reduction or loss of sense of smell. The majority of cases of CRS are treated with medical therapy consisting of topical steroids and nasal douching [3]. Antibiotics such as clarithromycin or amoxicillin may be used at the outset of therapy or for acute exacerbations of disease. In cases of CRS that are recalcitrant to medical treatment, surgical techniques may be warranted to improve the drainage pathway of the sinuses and to remove polyps and obstructive mucin. Non-invasive surgical interventions, known collectively as functional endoscopic sinus surgery (FESS), are now widely used, although there is limited evidence to support their efficacy [4]. The successful treatment of CRS is hampered by the heterogeneity of the disease. Chronic rhinosinusitis is a spectrum of diseases with a variety of causes or contributing factors including smoking, allergies, underlying systemic diseases, invasive or non-invasive fungal infections, viruses and bacteria [2], [5], [6]. Increasingly, it is becoming clear that microbial biofilms are associated with many cases of CRS. The growth of microorganisms within biofilms presents unique problems for the management of CRS. Within biofilms, micro-organisms are up to 1,000-fold more resistant to antibiotics than free-living cells of the same species [7]. Increased resistance is due to many factors, including the presence of a viscous polymeric matrix that restricts the penetration of antimicrobials, slow growth of bacteria, resistant phenotypes, and altered chemical microenvironments [8]–[11]. In keeping with these observations, there is limited evidence that either topical or systemic antibiotics improve the outcome of CRS infections [12], [13] underlining the need for new therapeutic approaches.

The role of biofilms in the initiation of CRS and their recalcitrance to treatment has received a great deal of attention over the last few years. A recent review of the literature identified 11 studies reporting the analysis of biofilms on sinus mucosa in CRS patients [14]. Several different techniques were employed to visualise biofilms. Arguably the most convincing method was fluorescence *in situ* hybridization, since this provides contrast between bacterial DNA and host cells. Biofilms on mucosal surfaces appear as punctate staining (bacterial cells), occasionally with some diffuse coloration, suggestive of extracellular nucleic acids [9]. All studies detected biofilms in a proportion of CRS patients, with prevalence varying from 25% to 100%. In contrast, only three of the eight studies that also analysed non-CRS controls identified biofilms on the non-CRS sinus mucosa, and these involved small numbers of patients. Collectively, these data clearly point to an association between biofilms on sinus mucosa and CRS. In addition, biofilms or biofilm-forming bacteria have been associated with unfavourable outcomes following FESS. For example, the presence of biofilms on paranasal sinus mucosa was correlated with persistent mucosal inflammation and requirements for lengthy post-surgical follow-up periods [15]. In a large cross-sectional study involving 518 CRS patients, the presence of sinus biofilms was significantly correlated with prior experience of sinus surgery, indicating either that biofilms contribute to CRS recurrence, or that FESS may promote the formation of biofilms [16]. Further, the capacity of paranasal sinus isolates of *Staphylococcus aureus* or *Pseudomonas aeruginosa* to form biofilms *in vitro* has been associated with the recurrence of CRS symptoms in the 12 month period following FESS [17].

There is strong evidence that the microflora of the maxillary sinus changes during acute sinusitis, towards a predominance of *Streptococcus pneumoniae*, *Haemophilus influenzae* and, less frequently, *Moraxella catarrhalis* [18], [19]. However, the microbial population present in CRS more closely resembles that in non-inflamed paranasal sinuses. The most common organisms both in CRS and in healthy patients include *Staphylococcus aureus*, coagulase-negative staphylococci, α-haemolytic streptococci, *Corynebacterium* spp. and strict anaerobes such as *Prevotella* spp., *Peptostreptococcus* spp. and *Propionibacterium* spp. [19]–[22]. Therefore, the development of biofilms within the paranasal sinuses, and the subsequent host responses to biofilms, may be more important for the pathogenesis of CRS than the *de novo* colonization of the upper respiratory tract by specific pathogens.

In light of the resistance of biofilm bacteria to conventional antibiotics, a number of novel approaches for treating biofilms have been proposed, including interfering with chemical communication between micro-organisms or degrading the biofilm matrix with enzymes [23], [24]. Biofilms associated with CRS are extremely heterogeneous, with many different organisms playing a role, and therefore any strategy to clear biofilms would need to target a component that is widely utilised by different microorganisms in the biofilm matrix. One possible target that has received significant interest in recent years is extracellular DNA (eDNA). The matrices of a many different bacterial and fungal biofilms contain eDNA, and this molecule has been shown to serve several critical functions including stabilising the biofilm structure [25]–[28], enhancing initial adhesion to surfaces [29], [30], promoting the exchange of genetic information [31], and acting as a nutrient store that can be utilised during nutrient depletion [32]. Recently, we have identified an extracellular bacterial deoxyribonuclease, NucB, from a marine isolate of *Bacillus licheniformis* strain EI-34-6 that can disperse biofilms by degrading eDNA [33]. The exogenous addition of NucB to biofilms formed by *Escherichia coli*, *Bacillus subtilis* or *Micrococcus luteus* resulted in almost complete removal of bacterial cells from surfaces. Hence, NucB has the potential to remove biofilms formed by Gram-positive or Gram-negative bacteria. It is anticipated that the development of new methods for disrupting viscous biofilm matrices will improve the post-surgical outcomes of FESS. In addition, such approaches may facilitate the surgery itself if the obstructive mucin is also targeted. This study therefore aimed to characterise the potential of NucB to disperse biofilms formed by microorganisms associated with CRS.

## Materials and Methods

### Ethics Statement

Ethical approval for the study was granted by the National Research Ethics Service Committee (North East – Sunderland) and each patient gave informed consent before enrolment.

### Collection of Specimens

A total of 20 patients undergoing FESS for the treatment of CRS at the Freeman Hospital, Newcastle upon Tyne, were recruited to this study. All patients met the CRS diagnosis criteria published by the Chronic Rhinosinusitis Task Force [2]. Patients were recruited to the study only if obstructive mucin was observed during the surgical procedure. During FESS, obstructive mucin that was dislodged surgically was collected with mucous traps (Sigma Aldrich) and immediately placed into sterile reduced transport fluid (RTF) [34]. Specimens were transferred to the laboratory and stored at 4°C. All samples were processed within 24 h.

### Transmission Electron Microscope Analysis of Obstructive Mucin

Samples of obstructive material removed from patients during FESS were cut into ∼1 mm^3^ pieces and placed into 2% glutaraldehyde immediately after surgery. These samples were dehydrated through a series of ethanol washes, embedded and sectioned at Electron Microscopy Research Services, Newcastle University. Sections were analysed in a transmission electron microscope (Philips, CM100).

### Visualisation of Bacteria on the Surface of Sinus Mucosa

Fluorescence *in situ* hybridisation (FISH) was performed using a peptide nucleic acid (PNA) probe corresponding to the well-characterised EUB338 probe [35]. The probe was synthesized as a fluorescein amidite (FAM) conjugate by Panagene. Mucosal biopsy specimens were fixed in 10% formalin directly after surgery, and stored at 4°C for up to one month. For PNA-FISH analysis, specimens were transferred to 50% ethanol and incubated for 16 h at −20°C. Biopsy material was transferred to 1 ml permeabilization buffer (10 mg/ml lysozyme in PBS) and incubated at 37°C for 30 min. Samples were immersed in 1 ml pre-warmed wash buffer (10 mM Tris-HCl pH 9.0, 1 mM EDTA) for 30 min at 55°C. Pre-warmed hybridization buffer (25 mM Tris-HCl pH 9.0, 100 mM NaCl, 0.5% SDS, 30% formamide) containing 150 pmol per ml of the PNA probe was added to samples and incubated in darkness for 90 min at 55°C. Unbound PNA probe was removed by incubating in pre-warmed wash buffer for 30 min at 55°C. Eukaryotic cells were counterstained by immersing the specimens in 1 ml PBS containing 2 µg ml^−1^ 4′,6-diamidino-2-phenylindole (DAPI) in darkness at 20°C for 15 min. Samples were glue-mounted onto a plastic surface and immersed in 2 ml PBS. Visualisation of surface bacteria and eukaryotic cells was performed using a Leica TCS SP2 microscope with an argon/neon laser for imaging FAM conjugates (excitation 495 nm, emission 520 nm), and DAPI (excitation 358 nm, emission 461 nm). Images were converted into z-stacks using Image J software [36].

### Isolation and Culture of Micro-organisms

A variety of growth media were employed for the isolation and routine culture of micro-organisms. Blood agar contained (per litre) 37 g Brain Heart Infusion (Oxoid), 5 g Yeast Extract (Merck), and 15 g Bacteriological Agar. After sterilization, 5% (v/v) defibrinated horse blood (TCS Biosciences) was added. Chocolate agar was prepared using the same recipe except that, after the addition of horse blood, the medium was heated to 70°C for 10 min. Fastidious Anaerobe Agar (FAA) was purchased from LabM and Sabouraud Dextrose Agar was from Oxoid.

For isolation of micro-organisms, a portion of sinus aspirate from each patient was homogenized in sterile phosphate buffered saline (PBS) and inoculated onto blood agar, chocolate agar, FAA and two plates of Sabouraud Dextrose agar. Blood and chocolate agar plates were incubated in 5% CO_2_ at 37°C. Pre-reduced FAA plates were incubated at 37°C anaerobically (Ruskinn, Bugbox Plus) in a gas mix consisting of 10% CO_2_, 10% H_2_ and 80% N_2_. The Sabouraud Dextrose agar plates were incubated aerobically, one at 37°C and the other at 30°C. Plates were examined every 24–48 hours for at least seven days. Individual colonies were picked and sub-cultured three times to obtain pure isolates. Strains were stored at −80°C in BHY medium [Brain Heart Infusion 37 g/L (Oxoid) and Yeast Extract 5 g/L (Merck)] diluted to 50% strength by the addition of glycerol.

### Identification of Isolates

All isolates were initially characterized by Gram staining, inspection of colony morphology and testing for catalase production, haemolysis and ability to grow aerobically or anaerobically. A single thick streak of each isolate was plated onto DNase agar (Oxoid) to test for extracellular nuclease activity. Plates were incubated aerobically or anaerobically at 37°C for between 24–96 h. Once colonies had grown, plates were flooded with 4 ml of 0.1% (w/v) toluidine blue (Sigma) to highlight nuclease production. The majority of clinical isolates were further identified to species level using a Matrix Assisted Laser Desorption/Ionization Time-of-Flight (MALDI-TOF) Mass Spectrometer (Bruker, Microflex) [37]. Isolates were streaked onto blood agar, incubated under 5% CO_2_ or in the absence of oxygen, at 37°C for 24 h and transferred to the Pathology Department, Freeman Hospital, Newcastle upon Tyne, for identification.

In cases where MALDI-TOF analysis yielded ambiguous results, for example the majority of α-haemolytic streptococci, bacterial identification was confirmed by analysis of the 16S rRNA gene. Bacteria were cultured in BHY broth, and harvested by centrifugation at 4,000 *g*. Cells were resuspended in 150 µl spheroplasting buffer [26% (w/v) raffinose, 10 mM MgCl_2_, 20 mM Tris-HCl, pH 6.8] supplemented with 37.5 µg lysozyme (Sigma) and 50 U mutanolysin (Sigma), and incubated at 37°C for 30 min. Following incubation, DNA was extracted using the MasterPure™ Gram Positive DNA Purification Kit (Epicentre® Biotechnologies) in accordance with manufacturer’s instructions. The DNA was suspended in 25 µl elution buffer (10 mM Tris pH 8.5).

The gene encoding 16S rRNA was amplified from 2 µl of extracted DNA in a reaction that also contained 5 µl (25 pmol) each of oligonucleotide primers 0063F and 1387R [38], 25 µl ReddyMix Extensor PCR Master Mix (Thermo Scientific) and 13 µl dH_2_0. PCR reactions were run using a GeneAmp PCR System 9700 (Applied Biosystems) with steps as follows: denaturation at 94°C, 2 min, followed by 35 cycles of (i) 94°C, 10 sec, (ii) 55°C, 30 sec, (iii) 68°C, 1 min and a final elongation at 68°C, 7 min. PCR products were checked on a 1% agarose gel, and fragments of the expected size were sequenced by MWG Eurofins. Forward and reverse sequences were aligned and sequence matched using the Ribosomal Database Project website (http://rdp.cme.msu.edu/).

### Purification of *B. licheniformis* NucB

NucB was produced using a previously described method [33]. Bacterial strain *Bacillus subtilis* NZ8900 containing plasmid pNZ8901 was inoculated into 5 ml sterile Luria Bertani broth (Sigma) containing 5 mg l^−1^ chloramphenicol and cultured aerobically at 37°C for 18 h. The culture was adjusted to an optical density (OD_600_) of 1.0, and 100 µL were transferred to 10 ml of sterile LB containing chloramphenicol and incubated aerobically at 37°C for 3 h until OD_600_ ∼1.0 was reached. At this point, 5% v/v cell free supernatant of an overnight *B. subtilis* ATCC6633 culture was added to provide the subtilin required to induce NucB production, and this was incubated for a further 2.5 h. Cells were removed by centrifugation at 6000 *g* for 20 min and the supernatant was sterilized by passing through a 0.2 µm syringe filter. The concentration of NucB was estimated by comparison with bovine serum albumin standards on a SDS-PAGE gel. NucB was stored at 4°C for up to 3 months. NucB was purified from the supernatant using trichloroacetic acid (TCA) precipitation followed by Superose™ 12 gel filtration. Proteins in the active fractions were further concentrated by TCA precipitation again and analysed by SDS-PAGE. The concentration of protein was estimated by densitometry analysis, in comparison with standards of known concentration [33].

### Growth and Biofilm Formation by CRS Isolates

Planktonic growth kinetics in batch culture were measured in BHY broth. Stock cultures of CRS isolates (see above) were diluted in BHY to a starting OD_600_ of ∼0.1. Cultures were incubated at 37°C and at hourly intervals, 1 ml samples were removed and OD_600_ was determined. For biofilm assays, 8 µl of CRS isolate stock cultures (see above) were added to triplicate wells of a sterile polystyrene 96-well plate (Corning 3595) containing 200 µl BHY broth. The lid was replaced and plates were wrapped in parafilm and incubated without shaking aerobically at 37°C for 18 h. Following growth, 150 µl of the non-adherent planktonic cells were removed and transferred to a clean 96-well plate and the OD_600_ was read in a microplate reader (BioTek Synergy HT) to quantify growth in the planktonic phase. To quantify biofilm extent, 100 µl of 0.5% (w/v) crystal violet were added to each well and incubated at 20°C for 15 min. Wells were rinsed 3 times with 200 µl PBS. Residual crystal violet was dissolved in 100 µl 7% acetic acid and the *A*_570_ was read in a microplate reader (BioTek Synergy HT). Each assay was performed three times independently. To assess the efficacy of NucB for dispersing biofilms, biofilms were cultured as above, and washed three times with PBS. Purified NucB (3 µg ml^−1^) or PBS alone was added to wells and incubated for 1 h at 37°C. Remaining biofilms were quantified by staining with crystal violet.

### Growth and Visualization of Biofilms on Glass Surfaces

The effect of NucB on biofilm architecture was visualized by confocal laser scanning microscopy (CSLM) or by scanning electron microscopy (SEM), using biofilms cultured on glass surfaces. Sterile 13 mm diameter glass coverslips were placed in wells of a six-well tissue culture plate containing 3 ml BHY. Wells were inoculated with 50 µl of stock bacterial cultures and incubated statically in air at 37°C for 18 h. Coverslips were removed and rinsed three times with PBS, and 1 ml NucB (3 µg ml^−1^) or 1 ml PBS (control) was added and incubated for 1 h at 37°C. For CSLM, coverslips were inverted onto a rubber O-ring that had been placed on a microscope slide and filled with Live/Dead® *Bac*Light™ stain (Molecular Probes). Biofilms were examined using a Leica TCS SP2 confocal microscope with an argon/neon laser for visualisation of SYTO® 9 (excitation 485 nm, emission 519 nm), and propidium iodide (excitation 536 nm, emission 617 nm). For SEM, coverslips were fixed in 2% (v/v) glutaraldehyde at 4°C for 16 h. Specimens were rinsed twice in PBS and dehydrated through a series of ethanol washes as follows: 25% ethanol 30 min, 50% ethanol 30 min, 75% ethanol 30 min, and two washes for 1 h in 100% ethanol. Samples were dried in a critical point dryer (Bal-tec), mounted on aluminium stubs and sputter coated with gold at Electron Microscopy Research Services, Newcastle University. Biofilms were visualised using a scanning electron microscope (Cambridge Stereoscan 240).

### Extraction and Analysis of eDNA in Biofilms Formed *In vitro*

Bacterial isolates were cultured in 6-well tissue culture dishes (Greiner) containing 3 ml of BHY broth for 72 h aerobically at 37°C. During this time, broth was carefully removed every 24 h and replaced with fresh medium. At the end of the 72 h incubation, medium was removed and PBS (1.5 ml) was added. Biofilms were gently removed from the surface of the wells using a plastic tissue culture cell scraper. Cells from four wells were combined together, and eDNA was purified by a modification of the method of Kreth *et al*. [39]. Briefly, cells were mixed by vortexing for 20 s, and incubated at 37°C for 1 h in the presence of 5 µg ml^−1^ of Proteinase K (Sigma Aldrich). Cells were separated from the supernatant, containing eDNA, by centrifugation at 16,000 *g* for 2 min. Extracellular DNA in the supernatant was extracted with phenol:chloroform:isoamyl alcohol (25∶24∶1). Samples were centrifuged at 16,000 *g* for 5 min to separate the phases, and the aqueous phase was collected. DNA was precipitated by the addition of isopropanol. The DNA was pelleted by centrifuging at 16,000 *g* for 10 min, air dried, and re-suspended in 50 µl of 10 mM Tris pH 8.5. For intracellular DNA, pelleted cells were resuspended in 150 µl spheroplasting buffer, and DNA was purified using the method described above.

The concentration and purity of DNA in each fraction was determined using a NanoDrop spectrophotometer. In addition, double stranded DNA (dsDNA) was quantified using the PicoGreen dsDNA reagent (Molecular Probes) and comparing with standards of known concentration according to the manufacturer’s instructions. Intracellular and extracellular DNA was visualized by agarose gel electrophoresis.

## Results

### Microscopic Analysis of Obstructive Mucin and Mucosal Biopsies from CRS Patients

**Figure 1:**
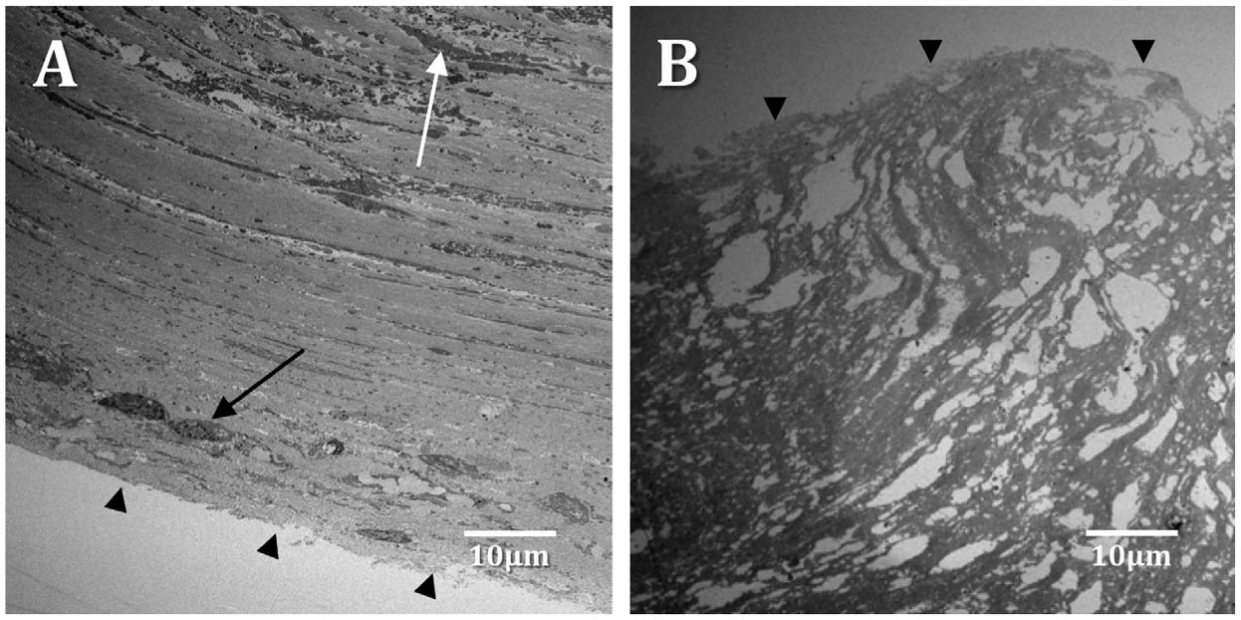
Transmission electron microscopy of obstructive mucin from CRS aspirates. In some cases (A), the mucin either formed a layered structure, with relatively intact cells towards the outer layers (black arrow) and more degraded cellular material further in (white arrow). (B) Alternatively, samples had little clear structure and the mucin was punctuated by pockets. The outermost layer of each sample is indicated by dark arrowheads.

Obstructive mucin is sometimes observed in the paranasal sinuses of CRS patients with or without polyps in the absence of other symptoms that would indicate fungal rhinosinusitis. The removal of this mucin from the paranasal sinuses is the cornerstone of surgical treatment of CRS [40]. This material is extremely tenacious and removing it significantly extends the length of time required in the operating theatre. To investigate the structure of the obstructive mucin, a portion of the material from two different CRS patients was cut into small pieces (∼1 mm^3^) immediately after surgery and fixed in glutaraldehyde. The mucin was embedded and sectioned for TEM analysis (see Materials and Methods). The material from the two patients appeared similar in structure, and consisted predominantly of an acellular matrix either with a striated appearance punctuated by occasional degraded host cells and cell debris (Figure 1A), or with little structure and many pockets (Figure 1B). Erythrocytes were sometimes observed (not shown). However, areas containing large numbers of eosinophils were not observed in any field of view. Additionally, bacterial cells and fungi were not seen within the matrix.

**Figure 2:**
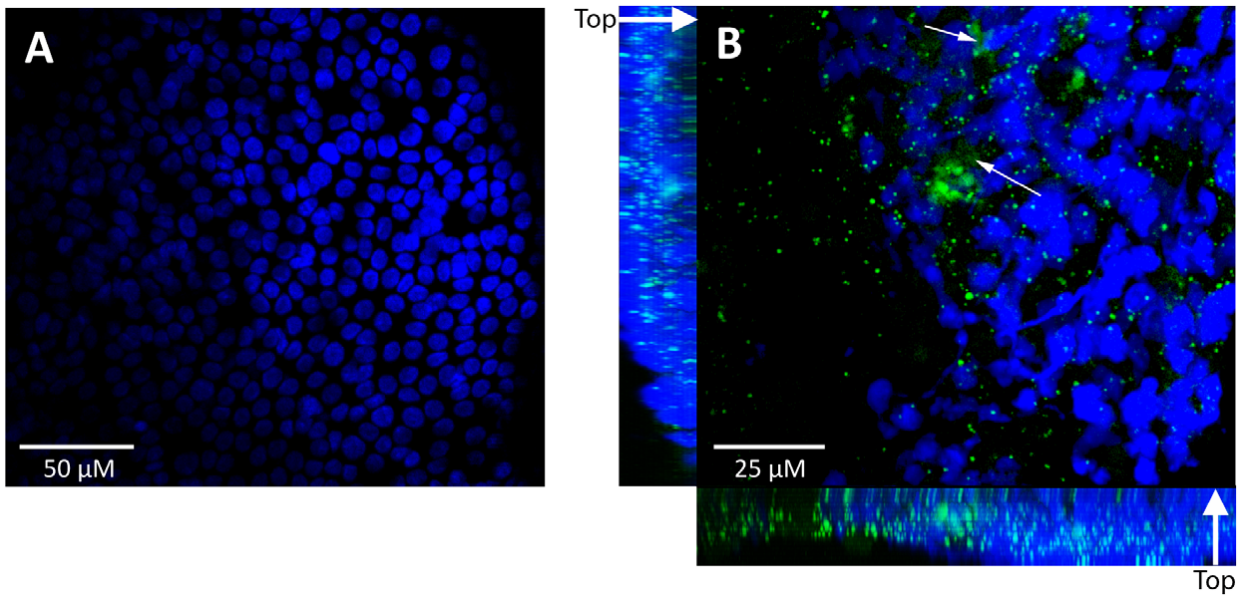
Confocal laser scanning microscopy of surface associated bacteria on mucosa removed from patients diagnosed with CRS. Bacterial DNA (green) was visualized using an EUB338 PNA-FISH probe, and epithelial cell nuclei (blue) were counterstained with DAPI. Maximum projection images are shown. In some fields, epithelial cells were observed in the absence of bacteria (A), and in other fields bacterial biofilm was evident (B). B includes *z*-stacks oriented from the outside of the mucosal biopsy specimen (labelled ‘top’) to the deeper layers (indicated by a thick white arrow). Small white arrows indicate patches of diffuse staining, consistent with the presence of extracellular nucleic acids.

The possibility that microbial cells were present but not directly observed could not be discounted. Therefore, mucosal biopsies were also collected from CRS patients, and analysed by confocal scanning laser microscopy (CSLM). Initially, propidium iodide was employed to stain bacteria. However, it was difficult to identify micro-organisms with confidence due to the lack of contrast between bacteria and host cells. Therefore, in subsequent samples bacteria were selectively stained by PNA-FISH with the EUB338 probe, and host cells were counterstained with DAPI. Using this approach, microbial cells were clearly identified on the mucosal surface (Figure 2). In *yz* and *xz* projections, it appeared that most of the micro-organisms were present in regions above the tissue surface or in a layer within the top 10 µm of the tissue. A three-dimensional representation of this image is shown in Figure S1. The EUB338 PNA probe targets bacterial 16S ribosomal RNA (rRNA) within metabolically active cells [41]. The detection of punctate staining with the EUB338 probe is indicative of microbial cells that were live prior to fixation. In addition to the sharp staining of cells, there were also patches of fluorescence from the PNA-FISH probe that were more diffuse (Figure 2B, arrows). Since PNA probes can hybridize with complementary DNA in addition to RNA [42], it is likely that this fluorescence represents extracellular microbial nucleic acids such as RNA or single stranded DNA.

### Isolation and Identification of Micro-organisms Associated with CRS

Overall, 75 strains of bacteria were isolated from obstructive mucin, comprising a total of 16 different genera and 32 separate species (Table 1). The most prevalent organism associated with CRS aspirates was *Staphylococcus epidermidis*, which was isolated from 15 of 20 specimens (75%). *Staphylococcus aureus* and *Streptococcus* spp. were each isolated from 7 patients (35% of samples), *Corynebacterium* spp. were isolated from 6 patients (30%), and *Propionibacterium* spp. from 5 patients (25%). Other organisms that were less frequently isolated included *Haemophilus influenzae*, *Moraxella catarrhalis*, *Neisseria* spp., *Finegoldia magna*, and *Enterobacter aerogenes*. The majority of isolated bacteria were facultative anaerobes. However, both obligate aerobes (for example, *Neisseria* spp. and *M. catarrhalis*) and obligate anaerobes (*F. magna*, *Propionibacterium* spp.) were also commonly isolated. No fungi were isolated from any of the patient specimens.

**Table 1:** Bacteria isolated from CRS aspirates.

Patient	Microbial Species Present^a^	Total number of isolates
**1**	*Enterobacter aerogenes, Staphylococcus epidermidis, Streptococcus* sp., *Streptococcus pneumoniae, Streptococcus salivarius*	5
**2**	*Haemophilus influenzae, Microccus luteus, Staphylococcus epidermis, Staphylococcus hominis, Streptococcus pneumoniae*	5
**3**	*Moraxella catarrhalis, * ***Staphylococcus aureus, Streptococcus anginosus***	3
**4**	*Klebsiella rhizophila, Staphylococcus epidermidis, * ***Streptococcus constellatus, Streptococcus intermedius, Streptococcus salivarius***	5
**5**	*Esherichia coli, * ***Staphylococcus aureus*** *, Staphylococcus epidermidis*	3
**6**	*Corynebacterium pseudodiphtheriticum, Finegoldia magna, Klebsiella pneumoniae, Moraxella catarrhalis, Staphylococcus epidermidis*, *Staphylococcus lugdunensis*	6
**7**	***Staphylococcus aureus*** *, Staphylococcus epidermidis, Staphylococcus warneri*, *Streptococcus constellatus*	4
**8**	*Corynebacterium propinquum, Staphylococcus epidermidis*, *Staphylococcus lugdunensis*	3
**9**	*E. aerogenes, Finegoldia magna, Propionibacterium* sp., *Streptococcus pneumoniae*	4
**10**	*Haemophilus influenzae, Moraxella catarrhalis, Staphylococcus epidermidis*, *Streptococcus pneumoniae*	4
**11**	*Staphylococcus epidermidis, Staphylococcus warneri*	2
**12**	*Citrobacter koseri, Staphylococcus epidermidis, Propionibacterium* sp., *Pseudomonas aeruginosa*	4
**13**	*Corynebacterium pseudodiphtheriticum, Propionibacterium granulosum, * ***Staphylococcus aureus***	3
**14**	*Staphylococcus epidermidis, Staphylococcus pasteuri, Staphylococcus warneri*	3
**15**	*Staphylococcus epidermidis, * ***Staphylococcus lugdunensis*** *, Propionibacterium acnes, Propionibacterium granulosum*	4
**16**	*Corynebacterium propinquum, Neisseria meningitidis, * ***Staphylococcus aureus***	3
**17**	*Neisseria* sp., *Staphylococcus epidermidis, * ***Streptococcus anginosus*** *, Streptococcus parasanguinis, Streptococcus salivarius*	5
**18**	***Staphylococcus aureus*** *, Staphylococcus epidermidis*	2
**19**	*Corynebacterium pseudodiphtheriticum, Propionibacterium avidium, * ***Staphylococcus aureus***, *Staphylococcus epidermidis*	4
**20**	*Corynebacterium accolens, Corynebacterium pseudodiphtheriticum, Lactobacillus* sp.	3

a Strains highlighted in bold text produced extracellular deoxyribonuclease.

The production of extracellular DNase enzymes by clinical isolates was assessed using DNase test agar and staining with toluidine blue. In total, 13 of the 75 isolates (17%) produced extracellular DNase (Table 1). All *S. aureus* isolates produced extracellular DNase, and other producers were *Streptococcus anginosus* group (*S. anginosus*/*S. constellatus*/*S. intermedius*) strains (80% of strains), *Staphylococcus lugdunensis* (33% of strains) and *Streptococcus salivarius* (33% of strains). Extracellular nuclease producers were isolated from 11 out of 20 (55%) patients. In only two cases, more than one nuclease producing organism was isolated from the same patient sample.

### Efficacy of NucB against Biofilm Forming Isolates

Twenty-four bacteria, isolated from patient specimens, were grown in 96-well microtiter plates to assay for biofilm formation. Representative strains of all species that produced extracellular DNase were selected for these studies, along with a similar number of non-producing organisms. Following incubation for 20 h in microtitre wells, all isolates had grown in the planktonic phase to OD_600_>0.1 with the exception of three strains: *S. anginosus* FH19, *S. constellatus* FH21 and *S. pneumoniae* FH26 (Table 2). Nevertheless, all of these strains produced biofilms that were detectable by crystal violet staining. In fact, *S. pneumoniae* FH26 produced a relatively strong biofilm (*A*_570_ = 1.87). Growth rates of each strain in BHY medium were determined in planktonic cultures (Table 2). No correlation was seen between the maximum growth rate of strains and the capacity to form biofilms. Generally, there was extensive variation in the extent of biofilm formation between different species and between different strains of the same species. For example, *M. catarrhalis* FH3 produced a very weak biofilm (*A*_570_ = 0.77), whereas *M. catarrhalis* FH4 formed extensive biofilms (*A*_570_ = 2.78). Of the strains tested, *Streptococcus anginosus* FH19 produced the least abundant biofilms (*A*_570_ = 0.22). The mean extent of biofilm formation by non-nuclease producers (*A*_570_ = 1.51, S.E. 0.19, n = 15) was not significantly different from that of nuclease producers (*A*_570_ = 1.48, S.E. 0.32, n = 9).

**Table 2:** Biofilm formation and NucB sensitivity of selected isolates from CRS aspirates.

Strain	Planktonic Growth yield OD_600_ Mean (S.E.)	Doubling Time (min) Mean (S.E.)	Biofilm Growth *A*_570_ Mean (S.E.)	Nuclease Production^a^	Remaining Biofilm after NucB Addition (%)	P-value
*Corynebacterium propinquum* FH1	0.39 (0.06)	235 (11)	1.79 (0.78)	−	105	0.558
*Corynebacterium pseudodiphtheriticum* FH2	0.86 (0.24)	129 (2)	2.61 (0.43)	−	92	0.577
*Moraxella catarrhalis* FH3	0.39 (0.07)	232 (6)	0.77 (0.21)	−	127	0.349
*Moraxella catarrhalis* FH4	0.42 (0.14)	155 (5)	2.78 (0.21)	−	124	0.032
*Staphylococcus aureus* FH5	0.40 (0.11)	62 (3)	1.84 (0.34)	+	77	0.003
*Staphylococcus aureus* FH6	0.50 (0.08)	74 (0.02)	0.71 (0.12)	+	59	0.000
*Staphylococcus aureus* FH7	0.79 (0.26)	61 (3)	1.23 (0.22)	+	40	0.000
*Staphylococcus epidermidis* FH8	0.27 (0.09)	81 (6)	2.29 (0.41)	−	114	0.077
*Staphylococcus epidermidis* FH10	0.48 (0.06)	90 (1)	1.59 (0.22)	−	67	0.001
*Staphylococcus epidermidis* FH11	0.54 (0.13)	104 (4)	1.52 (0.24)	−	74	0.010
*Staphylococcus lugdunensis* FH12	0.74 (0.03)	73 (4)	1.16 (0.23)	−	49	0.001
*Staphylococcus lugdunensis* FH13	0.78 (0.05)	70 (0.2)	0.57 (0.10)	−	66	0.001
*Staphylococcus lugdunensis* FH14	0.78 (0.13)	73 (16)	0.53 (0.05)	+	69	0.001
*Staphylococcus warneri* FH15	0.59 (0.18)	72 (4)	0.89 (0.25)	−	126	0.005
*Staphylococcus warneri* FH17	0.88 (0.16)	65 (3)	2.40 (0.55)	−	90	0.319
*Streptococcus anginosus* FH18	0.16 (0.05)	54 (3)	1.16 (0.07)	+	34	0.000
*Streptococcus anginosus* FH19^b^	0.07 (0.00)	90 (15)	0.22 (0.02)	+	59	0.015
*Streptococcus constellatus* FH20	0.22 (0.04)	103 (28)	1.90 (0.39)	+	44	0.001
*Streptococcus constellatus* FH21^b^	0.04 (0.03)	ND^c^	0.31 (0.05)	−	39	0.001
*Streptococcus intermedius* FH22	0.19 (0.02)	67 (2)	3.07 (0.80)	+	46	0.000
*Streptococcus pneumoniae* FH26	0.07 (0.03)	56 (1)	1.87 (0.31)	−	123	0.585
*Streptococcus salivarius* FH27	0.20 (0.06)	37 (3)	0.99 (0.04)	−	92	0.240
*Streptococcus salivarius* FH28	0.32 (0.04)	111 (29)	2.67 (0.96)	+	66	0.002
*Streptococcus salivarius* FH29	0.23 (0.03)	39 (0.7)	1.08 (0.04)	−	96	0.692

a Production of nuclease was measured on DNase agar, and is indicated by a ‘+’ sign.

b Isolates grew poorly in both the planktonic and biofilm phase.

c ND, not determined.

To assess the importance of eDNA in maintaining the structural integrity of biofilms, pre-formed biofilms were incubated for 1 h in the presence of the microbial DNase NucB (Table 2). Biofilms formed by 9 out of 9 (100%) nuclease producing strains were significantly reduced by NucB (T test comparing NucB treatment with buffer control, *p*<0.05, n = 3). By contrast, only 5 out of 15 (33%) of the biofilms produced by non-nuclease producing bacteria were dispersed by NucB. In addition, 2 out of 15 (13%) non-nuclease producers had slightly increased levels of biofilm following incubation with NucB than without the enzyme. To assess whether NucB had detrimental effects on the cells themselves, four different isolates, *S. aureus* FH7, *S. constellatus* FH20, *S. salivarius* FH29 and *M. catarrhalis* FH4, were cultured to mid-exponential phase in THYE broth and challenged with 5 µg ml^−1^ NucB. These isolates were selected as representative Gram-positive and Gram-negative organisms to assess the general toxicity of NucB for bacterial cells. Since the production of extracellular nucleases by bacteria is widespread, it seemed unlikely that DNase activity itself would be toxic to bacteria. Nevertheless, it was important to assess whether the NucB protein could inhibit the growth of bacteria. No effects were observed on the growth rate of cells following the challenge (data not shown). The number of viable cells in each culture continued to increase following NucB addition, and 1 h after adding NucB there was no difference in the number of viable cells in cultures containing NucB compared with control cultures without the enzyme. Overall, these data suggest that eDNA is an important component of the EPS for over 50% of the CRS isolates, including strains that produce extracellular DNase enzymes, and that addition of NucB dislodges cells without killing or inhibiting bacteria.

### Microscopic Analysis of *in vitro* Grown Biofilms

**Figure 3:**
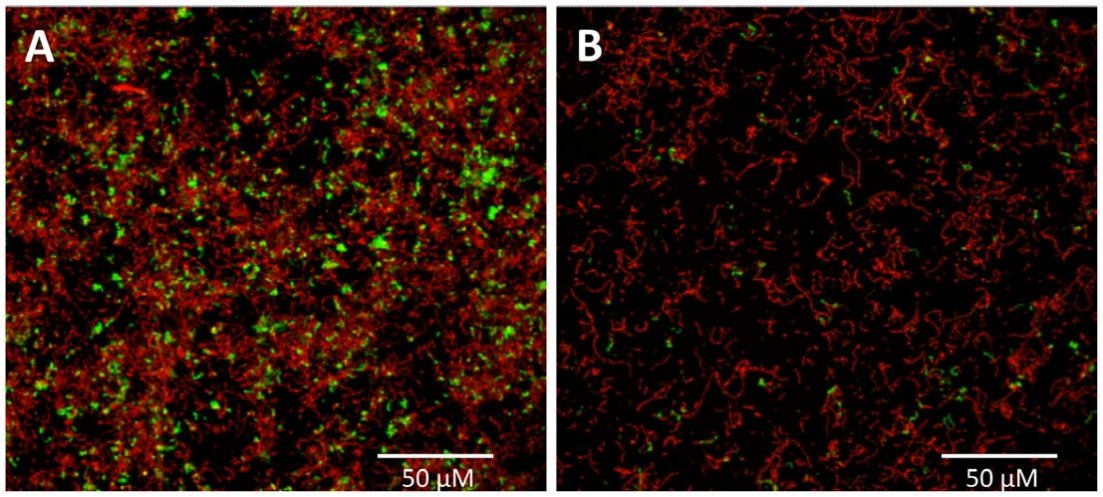
Confocal laser scanning microscopy of *Streptococcus constellatus* FH20 **biofilms with or without NucB treatment.** Biofilms were formed on glass surfaces and were visualised with CLSM using BacLight LIVE/DEAD stain, which stains compromised (dead) cells red and live cells green. (A) Biofilms treated with buffer alone, and (B) biofilms treated with NucB.

**Figure 4:**
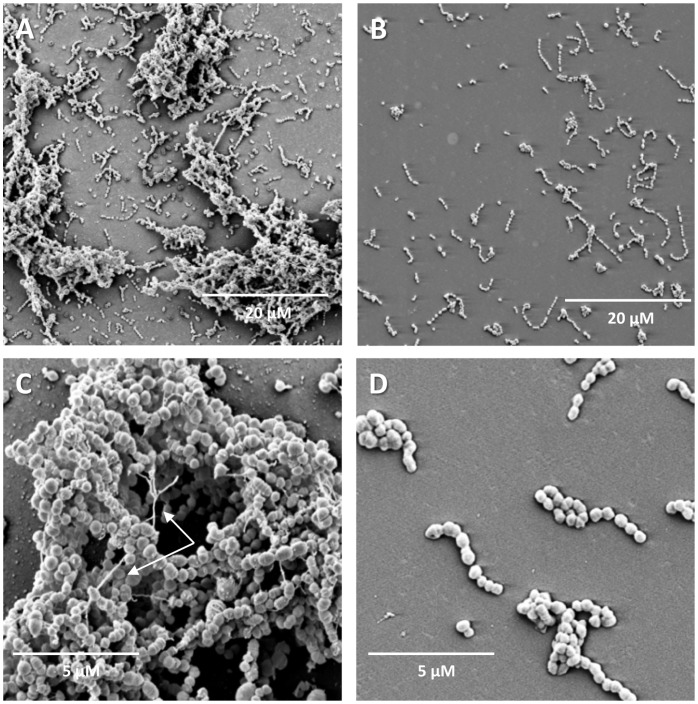
Scanning electron microscopy of *Streptococcus constellatus* FH20 biofilms treated with NucB or buffer control. Biofilms were visualised with SEM after treatment for 1 h with buffer (A) or with NucB (B). At higher magnification, extracellular material (white arrow) was observed in the absence of NucB treatment (C), but was not seen in NucB-treated biofilms (D).

To obtain more detailed information about the effects of NucB, biofilms of selected organisms were cultured on glass coverslips and analysed by CLSM and SEM. This work focussed on staphylococci and streptococci, since these were the genera most commonly isolated from CRS patients. In the absence of NucB treatment, biofilms formed by *S. constellatus* FH20 were relatively thin and consisted primarily of a single cell layer that covered most of the surface (Figure 3). In places, clusters of cells projected from the surface to a depth of ∼12 µm. Using BacLight Live/Dead stain, both live cells (green) and dead cells (red) were observed in biofilms. Biofilms that had been treated with NucB were clearly less extensive than the untreated controls, and consisted of sparsely distributed single cells or very small aggregates of <10 cells (Figure 3B). To obtain higher resolution images, similar biofilms were analysed by SEM (Figure 4). Again, in the absence of NucB, cell aggregates were evident and a relatively large proportion of the surface was covered by micro-organisms (Figure 4A). By contrast, NucB-treated biofilms almost exclusively contained isolated cells or small clusters of cells (Figure 4B). In addition, extracellular material was apparent in untreated biofilms under high resolution SEM (Figure 4C), that was not seen in biofilms incubated with NucB (Figure 4D). Biofilms formed on glass surfaces by *S. aureus* FH7 or *S. intermedius* FH22 were also visualised by SEM (not shown). As with *S. constellatus* FH20, biofilms that were treated with NucB contained far less biomass than those incubated in buffer alone. However, extracellular polymers were not observed in these organisms.

### Quantification of eDNA in Model Biofilms

**Figure 5:**
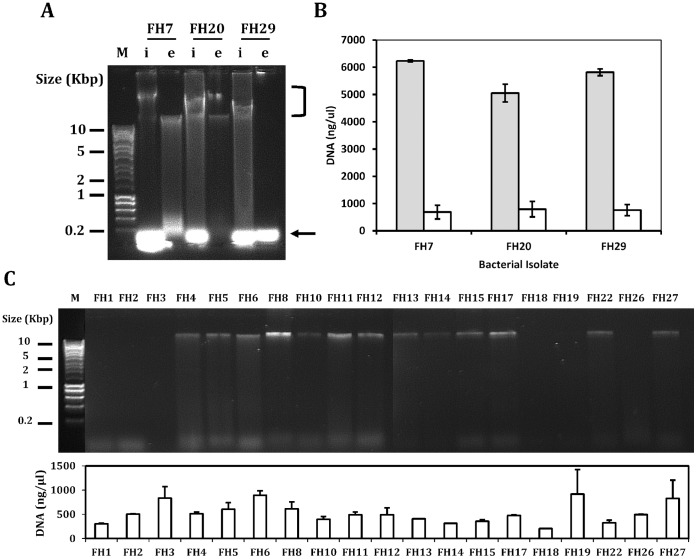
The visualization and quantification of eDNA from CRS isolates. (A) Intracellular DNA (i) or eDNA (e) was purified from bacterial biofilms of *S. aureus* FH7, *S. constellatus* FH20 or *S. salivarius* FH29, and analysed by agarose gel electrophoresis. High molecular weight chromosomal DNA is indicated by a black bracket; low molecular DNA or RNA is highlighted at the bottom of the gel by a black arrow. M; size marker. (B) The concentration of DNA in the intracellular (grey bars) and extracellular (white bars) fractions from bacterial biofilms was measured by NanoDrop spectrophotometry. Bars represent means of three independent extracts, and SEs are indicated. (C) Extracellular DNA concentration in biofilms was also visualised for another 19 isolates (see Table 1 for species names). In many cases, distinct bands were observed with an apparent migration at approximately 30 kbp. The total DNA concentration was measured by NanoDrop spectrophotometry, bars represent the average of three replicates and error bars are S.E.

To quantify levels of eDNA in model biofilms, eDNA and intracellular DNA (iDNA) was extracted from biofilm cultures of *S. aureus* FH7, *S. constellatus* FH20 and *S. salivarius* FH29. The eDNA was analysed by agarose gel electrophoresis (Figure 5). Sharp bands migrating at an apparent size of 30 kbp were observed in eDNA fractions of *S. aureus* FH7 and *S. constellatus* FH20. However, no high molecular eDNA bands were seen in *S. salivarius* FH29. Intracellular DNA from all three organisms appeared as a smear of high molecular weight fragments, probably due to binding of chromosomal DNA to cell wall fragments. In addition to the high molecular weight fragments, small fragments of DNA or RNA were seen at the bottom of the gel. Nucleic acids in each fraction were quantified using the Nanodrop spectrophotometer (Figure 5B). For each strain, eDNA represented approximately 5–10% of the total DNA present in the biofilm. To account for the possibility that samples may have contained RNA in addition to DNA, nucleic acids were also quantified using PicoGreen dye, which is strongly selective for double stranded DNA. No significant differences were observed between the total amount of eDNA in *S. salivarius* FH29 biofilms and eDNA in biofilms formed by the other two strains. Therefore, despite the lack of a clear band by agarose gel electrophoresis, it appears that eDNA was present in *S. salivarius* FH29 biofilms.

To assess whether eDNA was present in biofilms formed by other CRS isolates, biofilms of each strain were cultured in 6-well plastic dishes and eDNA was purified as described in the Methods section. Only two strains were omitted from this analysis: *S. constellatus* FH21 grew very poorly in biofilms and it was not possible to extract eDNA, and *S. salivarius* FH28 was prone to contamination and, after several attempts, it was decided not to pursue DNA purification from this strain. By agarose gel electrophoresis, sharp bands corresponding to high molecular weight eDNA products were observed in all *Staphylococcus* spp., *S. constellatus* FH20 and in *S. intermedius* FH22 (Figure 5C). By contrast, similar bands were not detected from *Corynebacterium* spp., *S. anginosus* or *S. pneumoniae*. Only *M. catarrhalis* and *S. salivarius* had inter-strain differences in the production of eDNA. Thus, eDNA was not visualised in *M. catarrhalis* FH3 biofilm extracts, whereas eDNA was clearly present in *M. catarrhalis* FH4 (Figure 5C). Similarly *S. salivarius* FH29 did not produce a band of eDNA on an agarose gel, whereas a sharp band was seen in *S. salivarius* FH27. The concentrations of eDNA in samples were determined using the Nanodrop spectrophotometer. Concentrations of eDNA in the extracts ranged from 206 ng µl^−1^ to 917 ng µl^−1^ (Figure 5C). Interestingly, there appeared to be little correlation between the concentration of eDNA and the presence or absence of a band on the agarose gel. For example, *M. catarrhalis* FH3 produced one of the highest concentrations of eDNA, but no band on the gel. Conversely, the eDNA concentration from *S. intermedius* FH22 was just 325 ng µl^−1^ even though this strain clearly produced a band of eDNA on a gel. The production of an extracellular nuclease did not correlate with the presence of a band of eDNA on a gel. All strains of *S. aureus* (nuclease-positive) and *S. epidermidis* (nuclease-negative) produced clear bands of eDNA, for example. With the exception of *S. anginosus* FH18 and *S. anginosus* FH19, all strains that failed to produce a clear band of eDNA on an agarose gel were insensitive to NucB treatment.

## Discussion

There is mounting evidence that microbial biofilms growing within paranasal sinuses are a major factor in the pathogenesis of CRS [14]. Bacterial biofilms have been most commonly detected on the sinus mucosa, whereas fungi tend to be more easily detected within the sinonasal mucous. Fungal growth is often accompanied by mucous secretions containing large numbers of intact or degraded eosinophils, known as ‘eosinophilic mucin’ or ‘allergic mucin’ [43]–[45]. The eosinophils appear to migrate intact from the tissues, and degrade or degranulate upon reaching the mucin, possibly in order to target fungi growing within the mucin. Allergic mucin may be present in the absence of fungi [46]. In our experience, a number of patients present with thick, tenacious mucin obstructing the paranasal sinsues, but without other evidence of fungal rhinosinusitis. We hypothesized that bacterial biofilms may contribute to the pathogenesis of CRS in these patients. The aim of this study was to characterize the microflora in paranasal sinuses of patients with obstructive mucin, and to assess the potential of a novel deoxyribonuclease enzyme for degrading biofilms formed by isolated micro-organisms.

Initially, the structure of obstructive mucin was investigated using TEM. This material contained relatively small numbers of degraded host cells. Therefore, this structure appears to be different from eosinophilic mucin [44]. In addition, fungi were not observed either by high-resolution TEM of obstructive mucin or by culture. The role of fungi in CRS is currently unclear. Culture-based studies have reported very low rates of isolation of fungi from CRS samples [20], [22], whereas the direct microscopic analysis of eosinophilic mucin in some cases detects fungi in 100% of patient samples [45]. In the patient cohort analysed here, CRS appeared to be of a non-fungal aetiology. Bacterial cells were also not detected within the obstructive mucin. However, bacteria do not generally produce filamentous structures such as hyphae and can be difficult to detect in thin sections. By carefully examining the mucosal surfaces, bacterial biofilms could be observed. Bacteria appeared to be localised on top of the tissue or within the outer layer of epithelial cells. It is likely that the dehydration steps involved in sample processing for FISH would have compromised the outer barrier of the tissue, leading to an irregular surface and the appearance of bacterial nucleic acid staining in regions slightly below the surface of the tissue. Alternatively, a proportion of the bacterial cells may have been present within the host cytoplasm, as has previously been described for *S. aureus* [47]. Nevertheless, the structure of biofilms was consistent with those previously described in CRS patients [9]. In addition, more than one species of bacterium was isolated from each of the 20 samples tested.

In total, 75 strains of bacteria were isolated from 20 CRS patients. On the whole, the organisms identified in this study were very similar to those identified in previous culture-based investigations into the microflora of CRS patients. Thus, staphylococci (both *S. aureus* and coagulase-negative staphylococci) and α-haemolytic streptococci were the most commonly isolated organisms, in agreement with published reports [18], [20]–[22]. Corynebacteria were isolated from seven CRS patients, and several potential pathogens were identified, including *M. catarrhalis*, *Neissseria* spp. and *H. influenzae*. All of these species have been isolated from CRS cases. However, their contribution to disease pathogenesis is unclear [20], [22]. *Enterobacteriaciae* have been frequently isolated from CRS patients [20]–[22], and this group of organisms was represented here by *C. koseri*, *E. aerogenes*, *E. coli*, and *K. pneumoniae*. There has been some debate about the presence of obligate anaerobes in paranasal sinuses of CRS patients. Thus, Doyle *et al*. [22] did not isolate anaerobes from chronic ethmoid sinusitis, whereas Brook [21] found that obligate anaerobes formed the majority of the bacteria isolated from chronic maxillary sinusitis. It is possible that the maxillary sinuses provide a more conducive environment for the growth and survival of anaerobes than the ethmoid sinuses. Here, samples were collected from a mixture of maxillary, sphenoid and ethmoid sinuses, and obligate anaerobes (*Propionibacterium* spp. or *F. magna*) were isolated from 6 of the 20 patients. The micro-organisms isolated and identified in this investigation are representative of the culturable microflora common in CRS patients. Recently, it has been shown that culture-independent, pyrosequencing analysis of the CRS microflora identifies a very similar microflora to that found by culture, although pyrosequencing has greater sensitivity for detecting difficult-to-culture or low-abundance micro-organisms [48].

The capacity of micro-organisms to form biofilms is likely to be important for the colonization of paranasal sinuses. Several studies have now provided direct evidence that bacteria are commonly present in biofilms on sinus mucosa in CRS patients [14]. Nevertheless, little is known about whether these organisms are particularly well-suited to forming biofilms. The ability of biofilm bacteria on sinus mucosa to produce biofilms *in vitro* has been assessed by directly inoculating mucosal swabs into a Calgary biofilm device model [49]. *P. aeruginosa* biofilm-defective mutants (*sad-31* and *sad-36*) were separately set up in the model to set the threshold value, below which samples were designated as non-biofilm formers. Using these criteria, 28.6% of 157 sinus aspirate samples produced biofilms. However, this is likely to be a significant underestimate of the total biofilm-forming capacity of mucosal bacteria since the biofilm experiments used only Luria-Bertani broth incubated aerobically, and therefore anaerobic or fastidious micro-organisms would not have grown. The capacity of clinical isolates of *P. aeruginosa*, *S. aureus* and coagulase-negative staphylococci, isolated >1 year post-FESS treatment, to form biofilms has been assessed as a possible predictor of the long-term outcomes of treatment [17]. Over 50% of strains tested produced biofilms, and the ability of *P. aeruginosa* or *S. aureus* to form biofilms appeared to be correlated with a poor clinical evolution of disease. To the best of our knowledge, there have been no investigations into the biofilm-forming ability of bacteria freshly isolated from patients during CRS treatment. We aimed to establish whether isolated CRS bacteria form biofilms *in vitro* and, further, whether eDNA contributes to the integrity of the biofilm.

In total, 24 isolated strains were tested for biofilm formation in a microplate model system, and all strains produced biofilms to some extent. The ability to form biofilms was not closely related to the growth rate or yield in planktonic cultures. These data are in line with previous studies on *Listeria monocytogenes* or *Salmonella enterica* strains, which also found no correlation between the growth rate or yields of individual strains and their capacities to form biofilms in microplate model systems [50], [51]. Representative strains of many of the species found in this study have been shown to produce DNase I-sensitive biofilms including, for example, *S. aureus* [52], [53], *S. pneumoniae* [54], [55], *Neisseria* spp. [56], [57], *P. aeruginosa* [29] and *E. coli* [53]. We have recently identified a novel DNase enzyme, NucB, from a marine strain of *Bacillus licheniformis* that has potent anti-biofilm activity against a number of bacteria including *E. coli* and *M. luteus* [33]. This enzyme is smaller in size (∼12 kDa) than many other nucleases, including DNase I, and appears to be well adapted to breaking up bacterial biofilms even at low concentrations [33]. A key goal of this study was to establish whether freshly isolated CRS-associated bacteria produce biofilms that are sensitive to NucB. Overall, >50% of the strains tested produced biofilms that were reduced upon treatment with NucB. In fact, the vast majority of staphylococci (8 of 10 strains tested) and streptococci (6 of 9 strains) produced NucB-sensitive biofilms. In contrast, two *Corynebacterium* spp. and two *M. catarrhalis* strains made biofilms that were not removed by NucB. In one case the *M. catarrhalis* biofilm was slightly, but significantly, increased by NucB treatment. Whilst eDNA commonly promotes adhesion and biofilm formation by bacteria, in rare cases eDNA has been shown to inhibit bacterial settlement [58]. It is possible that eDNA may be inhibitory to *M. catarrhalis* adhesion and that NucB-mediated eDNA degradation would therefore promote adhesion by this organism. This hypothesis requires further investigation.

The production of extracellular DNase enzymes by bacteria may influence the structure of biofilms. For example, isogenic nuclease-deficient mutants of *S. aureus*, *Neisseria gonorrhoeae* or *Vibrio cholerae* form thicker biofilms than their wild-type progenitor strains [57], [59], [60]. However, using *in vitro* or *in vivo* models of catheter biofilms, Beenken et al. [61] found that the total number of viable cells in biofilms of the clinical osteomyelitis isolate *S. aureus* UAMS-1 was not affected by mutation in either of two extracellular nuclease-encoding genes. Therefore, it is not clear whether microbial nucleases contribute to the gross biofilm structure in clinically relevant situations. Production of extracellular DNase enzymes has been reported for several of the genera isolated here. *S. aureus* is well-known to produce DNases, and DNase production is often used as a phenotypic test to differentiate *S. aureus* from coagulase-negative staphylococci. However, the test must be interpreted with caution, since some coagulase-negative staphylococci such as *S. lugdunensis* can produce nucleases [62]. In fact, one of the *S. lugdunensis* strains isolated here was found to produce DNase. *Corynebacterium diphtheriae* and *N. gonorrhoeae* have also been reported to be able to produce extracellular DNase enzymes [57], [63], but DNases were not detected in any of the *Corynebacterium* spp. or *Neisseria* spp. identified in this study. Production of DNases is variable in α-haemolytic streptococci [64], and 5 of the 9 streptococci isolated here produced DNase activity. Representative strains of all species that produced nuclease were tested in biofilm assays. Interestingly, all 9 nuclease-producing strains made biofilms that were reduced by treatment with the exogenous addition of NucB. These data provide clear evidence that the ability of a strain to produce extracellular nucleases does not preclude the formation of biofilms that are stabilised by eDNA. The production of extracellular DNases is tightly regulated in bacteria. For example, in *S. aureus*, nuclease production is regulated by the stress response sigma factor B [59]. Within biofilms, nucleases may be produced at low levels or by only a small proportion of the cells.

Here, direct evidence for the presence of eDNA in biofilms formed by 22 CRS isolates was provided by extraction and quantification of eDNA. All strains produced significant amounts of eDNA that could easily be measured in the Nanodrop spectrophotometer. A more detailed analysis was conducted on three different CRS isolates, including two that produce nucleases (*S. aureus* FH7 and *S. constellatus* FH20). In *S. constellatus* FH20 biofilms, extracellular material was observed by SEM (Figure 4). Extracellular DNA purified from *S. aureus* FH7 and *S. constellatus* FH20 biofilms was visualised as sharp high molecular weight bands on agarose gels with an apparent migration similar to that of intracellular chromosomal DNA. However, eDNA from *S. salivarius* FH29 was not detected by this technique. Nevertheless, quantitative measures indicated that extracellular nucleic acids were present in *S. salivarius* FH29 biofilms. Interestingly, in contrast to *S. aureus* FH7 and *S. constellatus* FH20, *S. salivarius* FH29 biofilms were not sensitive to NucB. Therefore, it appears that *S. salivarius* FH29 does not rely on large fragments of eDNA to stabilise biofilms. A broader analysis of the CRS isolates identified six other strains that did not produce defined bands of eDNA when analysed on agarose gels. Of these, four strains were insensitive to NucB indicating that, like *S. salivarius* FH29, these strains do not utilise large eDNA fragments for biofilm stabilisation. The two strains of *S. anginosus* did not produce visible bands of eDNA on gels, even though both strains were sensitive to NucB. It is possible that eDNA from *S. anginosus* was partially degraded, to the point where it did not form a defined band on a gel, but was still present in sufficient quantities to be utilised for maintaining the biofilm structure.

Improving the surgical treatment of CRS requires new methods for controlling microbial biofilms in the paranasal sinuses. The data presented here demonstrate that many CRS-associated bacteria produce biofilms that can be reduced by treatment with a microbial nuclease NucB *in vitro*. Given the high prevalence of CRS, even a 50% reduction in the colonization of sinus mucosa by micro-organisms would be predicted to have significant clinical benefits on a population level. Of course, the current study has focussed on *in vitro* work and it is acknowledged that translating the findings to the clinic will require further investigations in animal models and ultimately in patients. Before this can be done, the safety of NucB for clinical use must be established. We are currently in the process of testing the safety of NucB with a view to conducting clinical trials in future. In addition, it would be interesting to determine whether matrix-degrading enzymes act synergistically with antibiotics to control biofilm growth since this would present additional therapeutic possibilities. Ultimately, the utility of DNase enzymes to aid the treatment of CRS will depend upon *in vivo* data. Nevertheless, we have shown that NucB has clear potential for the control of biofilms formed by clinically important strains of bacteria.

## Supporting Information

Figure S1 Three-dimensional rotation showing micro-organisms associated with the outer layer of a mucosal biopsy. Bacterial DNA was hybridized with the EUB338 PNA-FISH probe, and appears green in the image. Host cell nuclei were counterstained blue. Bacterial cells (punctate green staining) are seen interacting with cells on the surface of the biopsy.
(AVI)
